# Factors facilitating clinical application of and adherence to evidence-based healthcare among medical professionals attending national competitions in Taiwan: a study based on the decomposed theory of planned behaviour

**DOI:** 10.1186/s12909-022-03610-5

**Published:** 2022-07-15

**Authors:** Jung-Mei Tsai, Yu-Hung Wu, Shu Yu

**Affiliations:** 1 Department of Nursing, MacKay Memorial Hospital, Taipei, Taiwan; 2 Center for Evidence-Based Medicine, MacKay Memorial Hospital, Taipei, Taiwan; 3 College of Nursing and Health Sciences, Dayeh University, Changhua, Taiwan; 4 Department of Medicine, MacKay Medical College, No.46, Sec. 3, Zhongzheng Rd., Sanzhi Dist., New Taipei City, 25245 Taiwan; 5 Department of Dermatology, MacKay Memorial Hospital, Taipei, Taiwan; 6 School of Nursing, National Yang Ming Chiao Tung University, No. 155, Sec. 2, Linong Street, Taipei, 112 Taiwan

**Keywords:** Decomposed theory of planned behaviour, Evidence-based healthcare, Clinical application, Seven action stages (7A)

## Abstract

**Background:**

Implementing evidence-based healthcare (EBHC) to improve the quality of patient care is a key issue for physicians and nurses. One of the most effective activities for achieving this is the annual topic-oriented clinical application national competition in Taiwan. Hundreds of clinical issues have been presented in this competition. By using the decomposed theory of planned behaviour (DTPB), this study explored physicians’ and nurses’ behaviour and adherence to the clinical application of EBHC after participating in the competitions.

**Methods:**

We conducted a 3-month cross-sectional online survey using a structured questionnaire adapted from the original study of the DTPB to collect behavioural and intention-related data. We also used a model of seven action stages (from *aware of* to *adhered to*) to assess target behaviours. We targeted contestants of the EBHC competitions between 1999 and 2017 as study participants. Of 631 teams, 321 teams completed the questionnaire, representing a 49.5% response rate. We applied structural equation modelling to test model fit. Moreover, we executed multivariate logistic regression to identify potential predictors.

**Results:**

Of the respondents, 33.3% reportedly reached the final *adhered to* stage. The DTPB model exhibited a good fit to the observed data. All constructs (usefulness, compatibility, peer influence, superior influence, self-efficacy, resource facilitating conditions, attitude, subjective norms, behavioural control, and intentions) were positively associated with the target behaviours, except for ease of use and technology facilitating conditions. Furthermore, the study model explained the variance in the target behaviours (37.0%). Having managerial duties (odds ratio [OR] =2.03, 95% confidence interval [CI] =1.10–3.77), resource facilitating conditions (OR = 1.06, 95% CI = 1.01–1.11), behavioural control (OR = 2.21, 95% CI = 1.47–3.32), and intentions (OR = 1.96, 95% CI = 1.40–2.73) were significant predictors of the achievement of the *adhered to* stage.

**Conclusions:**

The study demonstrated the association between determinants of behaviour and clinical applications and factors influencing adherence to EBHC among competition participants. The adherence rate was not high after the competitions, and this may be improved by promoting certain factors associated with the target behaviours.

**Supplementary Information:**

The online version contains supplementary material available at 10.1186/s12909-022-03610-5.

## Contributions to the literature

- The adherence to clinical application of evidence-based healthcare is low (33.3%) among hospital-based elites after national competition.
- Managerial duties, behavioural intention, perceived behavioural control, and resource facilitating conditions were the positive predictors of adherence.
- The decomposed theory of planned behaviour and the seven actions model are useful tools to understand implementing clinical application of evidence-based healthcare.

## Background

Evidence-based healthcare (EBHC) is recommended as a core competency for health professionals to improve the quality of patient care. The principle of EBHC is to integrate the best research evidence for a clinical situation with patient preference and practitioner expertise for healthcare decision-making [1, 2]. Practising EBHC involves five circular steps encountered in clinical problems: (1) formulate an answerable question in the format of population, intervention, comparison, and outcomes; (2) acquire the most relevant research from literature databases; (3) critically appraise the acquired evidence; (4) apply the appraised findings in clinical decision-making; and (5) audit the effectiveness and efficiency of clinical outcomes.

A seven-stage model was proposed to conceptualise how new evidence emerges from the evidence hierarchical pyramid [3] and goes through the application pipeline [4]. The model shows a steady decline in ratings from the (1) *aware of*, (2) *accepted*, (3) *applicable*, (4) *able*, (5) *acted on*, (6) *agreed to*, and (7) *adhered to* stages [5–7]. The first two stages pertain to the recognition of the clinical problem. The third to fifth stages pertain to the discipline of clinical quality improvement actions. The sixth and seventh stages are related to compliance aid. This model may serve as a useful scale for measuring practitioner behaviour towards emerging evidence.

In Taiwan, one of the major implementation activities of EBHC is a national competition held by government or societies. In this competition, most teaching hospitals assemble teams to compete in the searching skills for best evidence and to demonstrate quality improvement projects based on the EBHC steps in their institutions [8, 9]. Many critical clinical issues have been presented. However, little is known about the impact on and adherence of medical professionals to these hundreds of projects after the competition.

Accordingly, the objective of this study was threefold: (1) understand the behaviour towards the clinical application of EBHC among physicians and nurses who had participated in EBHC competitions, (2) use an intention-based model to explain the behaviour, and (3) determine the predictors of the behaviour required to achieve the final *adhered to* stage.

## Methods

### Study framework

The decomposed theory of planned behaviour (DTPB) [10] was used in the investigation of the target behaviour in this study. The DTPB is a popular model based on many theories, such as the theory of planned behaviour [11], the technology acceptance model [12, 13], and innovation characteristic theory [14]. The DTPB model has been used in numerous studies [10, 15–17].

The DTPB postulates that a behaviour of volition is an action of both behavioural intention (BI) and perceived behavioural control (PBC). BI reflects the motivation towards a behaviour, and PBC refers to the perceptions of internal and external control over a behaviour. BI can be divided into three components: attitude (favourable or unfavourable feelings towards a behaviour), subjective norms (perceptions that an important person or group would support an individual to engage or not engage in a behaviour), and PBC.

A fixed set of salient structures can be analysed for these constructs under the DTPB. Perceived usefulness, ease of use, and compatibility are incorporated as components of attitudinal beliefs. Perceived usefulness reflects the belief that a behaviour will enhance the performance of potential adopters. Ease of use, as the opposite of complexity, represents the degree to which a behaviour is perceived to be easy to understand, learn, or operate. Compatibility describes the degree to which a behaviour fits with the existing values, previous experiences, and current needs of potential adopters. The DTPB stipulates that these three components have a positive influence on attitude towards a behaviour.

Regarding subjective norms, the DTPB states that the influences of referent groups can be divided into peer influences and superior influences in an organisational environment. Peer influences reflect the expectation from referents, such as colleagues or friends. Superior influences reflect the expectation from referents, such as higher-ranking staff.

For PBC, the DTPB stipulates three salient beliefs: the internal notion of self-efficacy, resource facilitating conditions, and technology facilitating conditions. Self-efficacy is related to the perceived ability or self-confidence to perform a behaviour. Resource facilitating conditions reflect the availability of resources required to engage in a behaviour, such as time, money, or other specialised resources; technology facilitating conditions reflect the technical compatibility issues that may impede a behaviour.

### Definitions of assessed behavioural components

The first and second goals of this study were to understand the behaviour of physicians and nurses towards the clinical application of EBHC. The DTPB-based constructs used in the study are defined as follows: usefulness reflects the extent of enhanced competence in patient care; compatibility refers to the degree to which the target behaviour is assimilated into existing care methods; ease of use refers to the degree of the perceived ease of performing the target behaviour; peer influences refer to the expectation from friends and co-workers; superior influences refer to the pressure from superiors to perform the target behaviour; resource facilitating conditions refer to resources in terms of computers, costs, and databases; and technical compatibility issues in technology facilitating condition refer to technical compatibility issues that are associated with changes in care methods.

The third goal of this study was to assess the behaviour of adherence based on the seven-stage model [4]. Each of the stages is defined as follows: *aware of* means that evidence is realised only through EBHC competitions; *accepted* indicates that the evidence is included in routine work after the establishment of team consensus; *applicable* means that the evidence is ready for application in local patient groups with an established workflow/guideline and the required medical venue (i.e. equipment and materials); *able* means that staff members are competent in executing the proposed evidence by using the established workflow/guideline and planning strategies to overcome potential obstacles; *acted on* means that the evidence is implemented starting with a proper patient group; *agreed* means that staff and patients consent to the evidence through a shared decision-making for feasible solutions; and *adhered to* means that the staff and patients are committed to implementing and adhering to the evidence routinely.

### Study design and participants

We conducted a cross-sectional field survey nationwide in Taiwan to collect data on behaviours towards the clinical application of EBHC evidence among physicians and nurses who had participated in the EBHC competition (under the category of clinical application) prior to June 2017. Three Taiwanese professional associations—the Joint Commission of Taiwan (JCT), the Taiwan Nurses Association, (TWNA), and the Taiwan Evidence-Based Nurses Association (TEBNA)—organised the EBHC competition during the study timeframe. A total of 288 (during 1999–2017), 152 (during 2000–2017), and 281 (during 2013–2017) nurses and physicians participated in the competitions organised by the JCT, TWNA, and TEBNA, respectively.

### Instrument development

We adapted the scale developed by the original DTPB study [10] to measure each of the constructs in the study framework. The scale comprises 35 questions containing 60 items. We adapted all items to the context of our study. All items were rated by a 7-point Likert scale with the extreme anchors ‘strongly agree/very important’ and ‘strongly disagree/very unimportant’. We invited five experts (two physicians who were also Taiwanese Evidence-Based Medicine Association board members, two nurse administrators who were also TEBNA board members, and a nursing professor with expertise in qualitative research and EBHC) to review the initial items. We calculated the scores of content validity index (CVI) for the ratings provided by expert reviewers. The CVI scores ranged from .87 (subjective norms) to .95 (resource facilitating conditions), indicating that all items were retainable [18]. We performed a pilot study involving 30 subjects to assess the initial quality of the instrument. We derived Cronbach’s alpha coefficients ranging from .61 (peer influences) to .93 (resource facilitating conditions), indicating that the items were homogenous to the corresponding scales [19]. The questions along with the psychometric properties for each construct included in this study were reproduced in the Additional file 1: Appendix Table A1. We also added a self-reported behavioural item and several demographic questions to the questionnaire. The behavioural item was also rated on a 7-point Likert scale with anchors ranging from 1 (‘aware’) to 7 (‘adhered to’) based on the seven-stage model [4].

### Data collection procedures

We conducted a 3-month online anonymous survey to collect data from potential subjects. We primarily used Google Forms supplemented by regular mail and e-mail. The contact information of potential subjects was collected from the organisers of the EBHC competitions.

### Statistical analysis

We used IBM SPSS Statistics for Windows (version 20.0) for our statistical analyses. This paper presents descriptive statistics as percentages, means, and variances. For inferential statistics, we performed *t* and Pearson’s *χ*^2^ tests to determine associations between factors in terms of demographics and the target behaviours. We assessed the scale reliability by using Cronbach’s alpha coefficient, with the reliability threshold being set to >.70. Furthermore, we derived a summated scale for each subscale by using the mean value of underlying items. We used multivariate logistic regression to determine the predictors of the *adhered to* stage. In addition, we executed structural equation modelling (SEM) by using LISREL8 [20] to perform confirmatory factor analysis (CFA) and path analysis with the observed data set. A chi-square test is inappropriate for testing model fit in SEM. Accordingly, we selected three goodness-of-fit indices (along with their corresponding cutoff criteria) for assessment: the adjusted goodness-of-fit index (AGFI, >.90), comparative fit index (CFI, >.95), and the root mean square error of approximation (RMSEA, <.05). In general, the AGFI depends on the sample size [21]. We considered a *p* value of <.05 as statistically significant.

## Results

### Demographic characteristics and distribution of action stages

We collected a total of 321 complete questionnaires, representing a response rate of 49.5% (321/721). Over half of the respondents join the EBHC competitions organised by the JCT (162/288, 51.9%), followed by those organised by the TEBNA (102/281, 32.7%) and TWNA (48/152, 15.4%). Regarding the participation count for the EBHC competitions, most of the respondents participated once (133/321, 42.6%), some participated twice (86/321, 27.6%), and others participated twice or more (93/321, 29.8%). In addition, the majority of the respondents were nurses (235/321, 75.3%) or had managerial duties (181/321, 58.0%). Table 1 presents a summary of the demographic characteristics. Concerning the distribution of action stages, 33.3% (104/321) of the respondents reportedly achieved the *adhered to* stage and 16.0% (50/321) reached the *acted on* stage. Table 2 provides a summary of the distribution of action stages.

**Table 1:** Demographic characteristics of respondents to the survey on clinical application of EBHC (*N* = 312)

Demographic characteristics	All (***n*** = 312)	Achieve ‘adhered-to’ stage		***χ***^***2***^	***p***
	n (%)	No (***n*** = 208)	Yes (***n*** = 104)		
		n (%)	n (%)		
Gender				0.92	0.337
Male	70 (22.4)	50 (24.0)	20 (19.2)		
Female	242 (77.6)	158 (76.0)	84 (80.9)		
Age (years)				8.36	^*^0.015
< 30	12 (3.8)	10 (4.8)	2 (1.9)		
30–40	84 (26.9)	65 (31.2)	19 (18.3)		
> 40	216 (69.2)	133 (63.9)	83 (79.8)		
Working experiences (years)				15.07	^***^0.001
< 10	40 (12.8)	34 (16.3)	6 (5.8)		
11 ~ 20	139 (44.6)	100 (48.1)	39 (37.5)		
> 20	133 (42.6)	74 (35.6)	59 (56.7)		
Academic degree				0.37	0.545
College or below	97 (31.1)	67 (32.2)	30 (28.8)		
Master or above	215 (68.9)	141 (67.8)	74 (71.2)		
Type of institution				1.88	0.171
Medical center	231 (74.0)	149 (71.6)	82 (78.8)		
Regional or lower	81 (23.1)	59 (28.4)	22 (21.2)		
Managerial duty				11.06	^***^0.001
Yes	181 (58.0)	107 (51.4)	74 (71.2)		
No	131 (42.0)	101 (48.6)	30 (28.8)		
Profession				0.55	0.458
Nurse	235 (75.3)	154 (74.0)	81 (77.9)		
Physician	77 (24.7)	54 (26.0)	23 (22.1)		
Specialty				16.99	^**^0.005
Internal	107 (33.9)	72 (34.6)	35 (33.7)		
Surgery	44 (14.6)	29 (13.9)	15 (14.4)		
Pediatric	35 (11.1)	28 (13.5)	7 (6.7)		
Critical care	44 (14.6)	21 (10.1)	23 (22.1)		
OBS & GYN	30 (9.1)	16 (7.7)	14 (13.5)		
Others	52 (16.7)	42 (20.2)	10 (9.6)		
Associations				9.87	^**^0.007
JCT	162 (51.9)	110 (52.9)	52 (50.0)		
TWNA	48 (15.4)	23 (11.1)	25 (24.0)		
TEBNA	102 (32.7)	75 (36.1)	27 (26.0)		
Competition times				17.53	^***^0.001
1 time	133 (42.6)	104 (50.0)	29 (27.9)		
2 times	86 (27.6)	56 (26.9)	30 (28.8)		
3 times or more	93 (29.8)	48 (23.1)	45 (43.3)		

*EBHC* Evidence-based healthcare

*JCT* Joint Commission of Taiwan, *OBS* Obstetrics, *GYN* Gynaecology, *TWNA* Taiwan Nurses Association, *TEBNA* Taiwan Evidence-Based Nurses Association

^*^p < .05; ^**^p <.01; ^***^p <.001

**Table 2:** Distribution of clinical application achievements in seven EBHC action stages among survey respondents (*N* = 312)

Action stage	N (%)	Accumulated achievement (%)
Aware of	43 (13.8)	100.0%
Accepted	15 (4.8)	86.2%
Applicable	39 (12.5)	81.4%
Able	18 (5.8)	68.9%
Acted on	50 (16.0)	63.1%
Agreed	43 (13.8)	47.1%
Adhered to	104 (33.3)	33.3%

*EBHC* Evidence-based healthcare, *N* Number

### Model fit and hypothesis assessment

For CFA, the hypothesised structure was a good fit to the data (*χ*^2^ = 539.89, *df* = 517, *p* = .24; RMSEA = 0.01; CFI = 1.00; AGFI = .89). The path analysis results revealed that the goodness-of-fit indices (RMSEA = .03; CFI = .99; AGFI = .86) were satisfactory, although *χ*^2^ was significant (*χ*^2^ = 746.79, *df* = 544, *p* < .001); this indicates that the study model exhibited a good fit to the observed data.

**Fig. 1:**
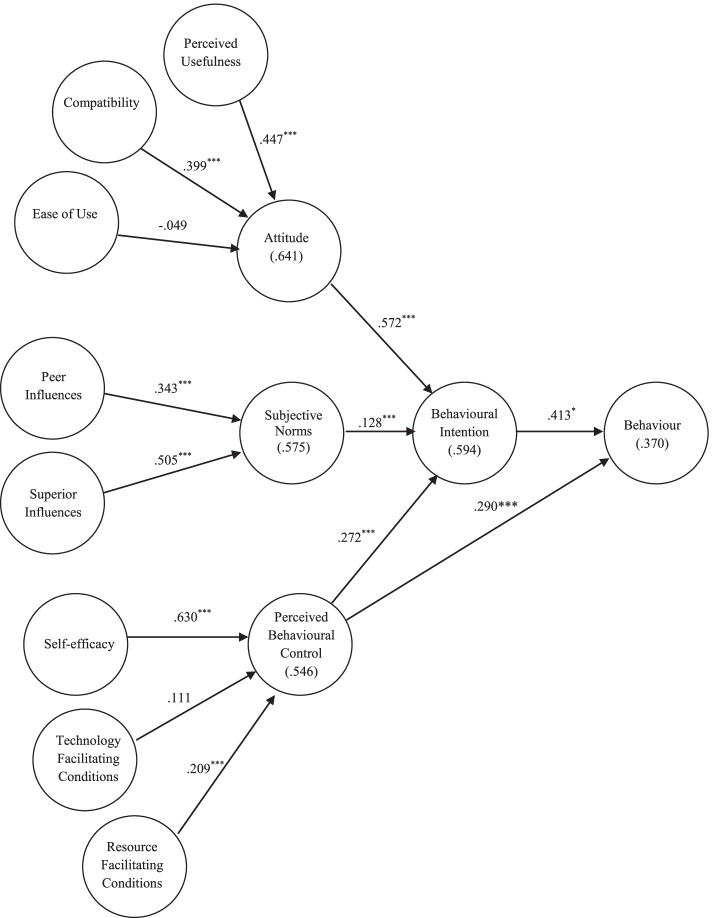
Study framework based on the decomposed theory of planned behaviour, along with path coefficients. ^*^p < .05; ^**^p < .01; ^***^p < .001; R^2^ values are provided in parentheses

Figure 1 illustrates the path coefficients and explanatory powers for the principal constructs of the DTPB. Perceived usefulness, compatibility, and ease of use jointly explained approximately 64.1% of the variance in attitude, and peer influence and superior influence explained approximately 57.5% of the variance in subjective norms. Furthermore, self-efficacy and the two facilitating conditions (resource and technology facilitating conditions) collectively explained approximately 54.6% of the variance in PBC. Overall, the model explained nearly 59.4% of the variance in BI and approximately 37.0% of the variance in target behaviours. As indicated in Fig. 1, the paths from perceived usefulness and compatibility to attitude were significant. However, the path from ease of use to attitude was non-significant. Both peer and superior influences were significantly linked to subjective norms; self-efficacy and resource facilitating conditions (i.e., time- and cost-related measures) were significant determinants of PBC. All three determinants of intention were significantly related to BI. Finally, both BI and PBC were significant determinants of behaviour.

Table 3 presents a summary of the total effects on BI and the target behaviours. As shown in this table, attitude, subjective norms, and PBC all had indirect effects on the target behaviours. In addition, perceived usefulness, compatibility, peer and superior influences, self-efficacy, and resource facilitating conditions all had indirect effects on the target behaviours. However, the indirect effects of ease of use and technology facilitating conditions were non-significant.

**Table 3:** Total effect of DTPB constructs on behavioural intentions and behaviours

Constructs	Behavioral intention	***t***	Behavior	***t***
	Standardized effect		Standardized effect	
Perceived usefulness	0.256	^***^3.541	0.106	^**^3.137
Compatibility	0.228	^**^3.249	0.094	^**^2.930
Ease of use	−0.028	−1.147	−0.012	−1.131
Peer influences	0.044	^*^2.396	0.018	^*^2.258
Superior influences	0.044	^*^2.396	0.027	^*^2.258
Self-efficacy	0.172	^***^5.068	0.253	^***^5.406
Technical facilitating conditions	0.030	1.767	0.045	1.780
Resource facilitating conditions	0.057	^**^2.845	0.084	^**^2.901
Attitude	0.572	^***^11.94	0.236	^***^5.891
Subjective norm	0.128	^**^2.818	0.053	^**^2.602
Perceived behavioral control	0.272	^***^6.139	0.402	^***^6.767
Behavioral intention			0.412	^***^6.606

*DTPB* Decomposed theory of planned behaviour

^*^p < .05; ^**^p < .01; ^***^p < .001

### Association of the adhered to stage with influential factors

As shown in Table 4, several factors in the demographic characteristics had preliminary effects on the target behaviours to achieve the *adhered to* stage. These included age (χ^2^ = 8.36, *p* < .05), work experience (χ^2^ = 11.06, *p* < .05), having managerial duties (χ^2^ = 11.06, *p* < .05), speciality (χ^2^ = 16.99, *p* < .05), association organising the EBHC competitions (χ^2^ = 9.87, *p* < .05), and EBHC competition count (χ^2^ = 17.63, *p* < .05).

**Table 4:** Predictors, related to demographic and DTPB constructs, of adherence

Predictors	Achieving the ‘adhered to’ stage				
	B	S.E.	***p***	OR	95% CI
Managerial duty (reference group: no)					
Yes	0.71	0.31	0.024	2.03	1.10–3.77
Behavioral intention^a^	0.67	0.17	0.000	1.96	1.40–2.73
Perceived behavioral control^a^	0.79	0.21	0.000	2.21	1.47–3.32
Resource facilitation conditions^a^	0.06	0.03	0.021	1.06	1.01–1.11
Constant	−10.26	1.28	0.000	0.00	

*DTPB* Decomposed theory of planned behaviour

^a^summated scale with the mean of the underlying items for the principal constructs in the study model

We performed logistic regression to further explore the predicting factors for the *adhered to* stage by using both the demographic items and the summated scales of the DTPB constructs. The values of the summated scale of a construct are presented as the means of underlying items. The logistic regression analysis results indicated that the achievement of the *adhered to* stage was determined by managerial duties (odds ratio [OR] = 2.03, 95% confidence interval [CI] = 1.10–3.77; *p* < .001), resource facilitating conditions (OR = 1.06, 95% CI = 1.01–1.11; *p* < .05), PBC (OR = 2.21, 95% CI = 1.47–3.32; *p* < .05), and BI (OR = 1.96, 95% CI = 1.40–2.73; *p* < .05).

## Discussion

This study demonstrated major factors affecting the positive behaviour of medical professionals in the clinical application of EBHC after competitions, including perceived usefulness and compatibility (in attitude), peer influences and superior influences (in subjective norms), and self-efficacy and resource facilitating conditions (in PBC). We observed that 33.3% of our survey respondents could achieve the final *adhered to* stage for their clinical healthcare. Four major factors—managerial duties, BI, PBC, and resource facilitating conditions—were the predictors of adherence.

### Strength and implications for practice and research

The study findings supported the hypotheses for the influence of attitude, subjective norms, and on BI. These findings are consistent with those of previous studies [9, 14–16]. The findings indicate that physicians and nurses were motivated by the competition and exhibited positive appraisals, reception of referential opinions, and control over competence and resources for the clinical application of EBHC. In terms of behaviour, the study findings supported the influence of BI and PBC on behaviour. The findings are in accordance with the original DTPB study [10].

Our results also reveal that perceived usefulness and compatibility had positive effects on attitude. These findings are consistent with those of other studies on healthcare [15–17] and the original DTPB study on computer centre usage [10]. The usefulness for enhancing competence in patient care and the compatibility to assimilate evidence-based care strengthened the attitudes towards the clinical application of EBHC. However, the influence of ease of use on attitude was non-significant. The association of ease of use with attitude was empirically unstable [17]. One possible explanation is that the participants in the competitions and the survey respondents were elite practitioners from their respective institutions; therefore, they were familiar with all EBHC steps, which affected the influence of this factor.

Our findings support the influences of peers and superiors on subjective norms, similar to earlier studies [10, 17]. This indicates that the respondents complied with the expectations of peers and superiors to apply EBHC. The compliance was also reflected on their perception of the desires of referent groups. Healthcare professionals typically work in organisational environments and interact in a team atmosphere in their daily routines when providing care to patients. This suggests that orienting leadership and team culture towards fostering subjective norms and peer/superior influences would be effective in this application.

Self-efficacy and resource facilitating conditions were significant predictors of PBC in this study, a finding that is consistent with those of earlier studies [10, 17]. Self-efficacy reflects the competence to apply EBHC. Resource facilitating conditions refer to resources provided by healthcare institutions. However, our results did not demonstrate technology facilitating conditions to be an antecedent of PBC. This suggests that the survey respondents were highly capable of handling the technical compatibility issues associated with changes in care methods based on EBHC.

Our logistic regression analysis results indicate that BI, PBC, and resource facilitating conditions in the DTPB constructs were all associated with adherence. Another major factor was the presence of managerial duties. Medical staff members with managerial duties tended to have stronger motivation and control of resources. They were more competent to adhere to new caring procedures. Therefore, the sharing of duties, responsibilities, and resources may enhance the adherence of physicians and nurses without managerial duties to the clinical application of EBHC.

Our finding that 33.3% of the respondents achieved the *adhered to* stage indicates the difficulty of implementing new healthcare procedures based on good and valid evidence. Although more than 80% of the respondents were aware of and accepted the applicable clinical issues, only 63.1% could turn evidence into real actions and less than 50% could execute it in their practice. The barriers might come from other team members who do not have a good concept of EBHC or from the lack of institutional support. Nevertheless, based our study results, we might provide support for factors affecting BI and PBC to encourage motivation and behavioural change. For example, resource facilitating conditions were an external drive that might help to achieve the adherence. Some well-functioned EBHC centres with experienced multidisciplinary teams attended the competitions more often. They continued to demonstrate improvement in healthcare quality among different medical fields.

### Study limitations

First, the questionnaire design required respondents to provide information on the topics of the EBHC competitions, which might have caused a hint and unwanted expectation. Subjects who cared about the consequences of the study or ceased to apply the topics may refuse to provide their responses. Second, because our study comprised only competition participants, the results may not be generalisable to other medical professionals. They were mostly chosen in their institution and were more capable to perform EBHC. This might explain why the effects of ease of use and technology facilitating conditions were not significant in this study. Third, approximately half of the research targets did not respond to the survey. Some of the non-responders might not be interested in EBHC, and the percentage of adherence pipeline might be even lower than the current results. Forth, there might be a recall bias due to a wide range of recalling times. However, the bias could be mitigated in the evaluation of behaviour of adherence, which reflected the current practice. Fifth, the study focused on individual factors and could not evaluate the systematic effect from institution or government policy. Finally, most of our respondents worked in medical centres (74.0%). Although not a statistically significant factor affecting adherence, this aspect of the study population may be important in promoting the clinical application of EBHC in local hospitals or through local practitioners.

## Conclusions

This study is the first large-scale survey to demonstrate factors affecting behaviour and adherence to EBHC after topic-oriented competitions in Taiwan. The DTPB provided a robust model to demonstrate the relationship between determinants of the behaviour and clinical application. The seven-stage model of EBHC application pipeline helped to understand the adherence of clinical implementation. Only 33% of the respondents could achieve the final *adhered to* stage. Through logistic regression, we identified several predictors pertaining to demographics and the DTPB principal constructs that may help improve the implementation of EBHC in clinical environments. Healthcare institutions could encourage their staff with managerial duties to attend the EBHC competitions and support related resources to maximize the adherence of clinical applications.

## Supplementary Information

**Additional file 1: Appendix Table A1.** DTPB questions adapted for EBHC CA survey.

## Data Availability

The datasets generated and/or analysed during the current study are not publicly available due to private work on doctorial thesis but are available from the corresponding author on reasonable request.

## Abbreviations

BI: Behavioural intention
CVI: Content validity index
DTPB: Decomposed theory of planned behaviour
EBHC: Evidence-based healthcare
JCT: Joint Commission of Taiwan
PBC: Perceived behavioural control
TEBNA: Taiwan Evidence-Based Nurses Association
TWNA: Taiwan Nurses Association
