# Supplementary material for: Factors facilitating clinical application of and adherence to evidence-based healthcare among medical professionals attending national competitions in Taiwan: a study based on the decomposed theory of planned behaviour
Source: BMC Med Educ. 2022 Jul 15;22:546. doi: 10.1186/s12909-022-03610-5 (PMC9284906; doi:10.1186/s12909-022-03610-5)
Supplement: Supplementary file 1 — Additional file 1: Appendix Table A1. DTPB questions adapted for EBHC CA survey. [file 12909_2022_3610_MOESM1_ESM.doc]

**Appendix 1**

**Table A1 | DTPB questions adapted for EBHC CA** survey

| **No.** | **Questions** | **Mean (SD)** |
| --- | --- | --- |
| **Perceived usefulness** (α =.869) | | 27.21 (7.87) |
| 1 | Using TEEHC will improve my ability to provide patient care. | 25.9 (11.58) |
| 2 | The TEEHC will be of no benefit to me. | 26.57 (10.84) |
| 3 | The advantages of the TEEHC will outweigh its disadvantages. | 27.93 (9.69) |
| 4 | Overall, using the TEEHC will be advantageous. | 28.43 (10.04) |
| **Compatibility** (α = .866) | | 29.02 (10.39) |
| 5 | Using the TEEHC will fit well with the way I work. | 27.4 (10.58) |
| 6 | Using the TEEHC will fit into my working style | 29.9 (12.81) |
| 7 | The setup of the TEEHC will be compatible with the way I work. | 29.77 (11.87) |
| **Ease of use** (α = .972) | | 24.09 (10.76) |
| 8 | The workflow of the TEEHC will be difficult to follow | 23.6 (11.36) |
| 9 | It will be difficult to learn how to use the TEEHC. | 23.57 (12.39) |
| 10 | It will be easy to operate the apparatus or equipment of the TEEHC. | 25.1 (13.02) |
| **Peer influences** (α = .656) | | 18.37 (7.48) |
| 11 | My friends would think that I should use the TEEHC in my work. | 17 (8.13) |
| 12 | My coworkers would think that I should use the TEEHC in my work. | 19.73 (9.44) |
| **Superior influences** (α = .930) | | 25.35 (11.85) |
| 13 | My boss would think that I should use the TEEHC in my work. | 25 (12.69) |
| 14 | I will have to use the TEEHC in my work because my boss requires it. | 25.7 (13.38) |
| **Self-efficacy** (α = .760) | | 23.59 (10.44) |
| 15 | I would feel comfortable using the TEEHC on my own. | 23.3 (11.24) |
| 16 | If I wanted to, I could easily use the TEEHC on my own. | 22.5 (11.85) |
| 17 | I would be able to carry out the workflow in the TEEHC even if there was no one around to show me how to do it. | 24.97 (11.31) |
| **Technology facilitating conditions** (α = .767) | | 26.38 (10.82) |
| 18 | The pieces of equipment/apparatus (or guidelines) for the TEEHC are not compatible with the ones I use now. | 28.3 (11.72) |
| 19 | The workflow in the TEEHC is not compatible with the one I use now. | 25 (11.19) |
| 20 | I will have trouble carrying out the TEEHC. | 25.83 (11.81) |
| **Resources facilitating conditions** (α = .783) | | 23.32 (10.75) |
| 21 | There will not be enough computers/equipment/materials for everyone to use the TEEHC. | 22.73 (10.68) |
| 22 | Using the TEEHC will be too expensive. | 24.1 (12.62) |
| 23 | I won't have the right database when I need it to use the TEEHC. | 23.13 (11.12) |
| **Attitude** (α = .978) | | 4.35 (1.56) |
| 24 | Using the TEEHC is a (bad/good) idea | 4.6 (1.793) |
| 25 | Using the TEEHC is a (foolish/wise) idea. | 4.2 (1.648) |
| 26 | I (dislike/like) the idea of using the TEEHC. | 4.4 (1.868) |
| 27 | Using the TEEHC would be (unpleasant/pleasant). | 4.2 (1.669) |
| **Subjective norms** (α = .912) | | 3.18 (1.39) |
| 28 | People who influence my behaviour would think that I should use the TEEHC. | 3.1 (1.626) |
| 29 | People who are important to me would think that I should use the TEEHC. | 3.27 (1.53) |
| **Perceived behavioural control** (α = .922) | | 2.73 (1.28) |
| 30 | I would be able to use the TEEHC. | 2.73 (1.461) |
| 31 | Using the TEEHC is entirely within my control. | 2.77 (1.633) |
| 32 | I have the resources *and* the knowledge *and* the ability to make use of the TEEHC. | 2.7 (1.442) |
| **Behavioural intention** (α = .953) | | 2.71 (1.31) |
| 33 | I might use the TEEHC for a while. | 2.73 (1.596) |
| 34 | I will use the TEEHC for a while. | 2.73 (1.461) |
| 35 | I will continue to use the TEEHC for a while. | 2.67 (1.446) |

DTPB: decomposed theory of planned behaviour; EBHC: evidence-based healthcare; CA: clinical application; SD: standard deviation; TEEHC: topic evidence presented in evidence-based healthcare competition.
